# Latissimus dorsi rotational flap combined with a custom-made scapular prosthesis after oncological surgical resection: a report of two patients

**DOI:** 10.1186/s12885-018-4883-7

**Published:** 2018-10-20

**Authors:** Giovanni Beltrami, Gabriele Ristori, Guido Scoccianti, Angela Tamburini, Rodolfo Capanna, Domenico Campanacci, Marco Innocenti

**Affiliations:** 1 Department of Paediatric Orthopaedic Oncology, Azienda Ospedaliero Universitaria Careggi, Largo Brambilla 3, 50134 Florence, Italy; 2 Department of Reconstructive and Oncologic Orthopaedics, Azienda Ospedaliera Universitaria Careggi, Florence, Italy; 3 Department of Orthopedic Oncology and Reconstructive Surgery, Azienda Ospedaliero Universitaria Careggi, Florence, Italy; 4 Department of Ortopaedics and Traumatology, Azienda Ospedaliero Universitaria Pisana, Pisa, Italy; 5 Department of Plastic Surgery and Microsurgery, Azienda Ospedaliero Universitaria Careggi, Florence, Italy

**Keywords:** Latissimus dorsi flap, Oncology, Orthopaedics, Scapular custom-made prosthesis, Scapulectomy, 3D printing

## Abstract

**Background:**

Sarcomas that arise from the scapula or periscapular soft tissues often require a total scapulectomy. This often implies a large complex tissue defect that needs adequate reconstruction of both bone and soft tissue. Although various methods have been developed, no optimal procedure has emerged. Postoperative complications are common and functional recovery is not always satisfactory. This study aims to present a new surgical technique that combines a custom-made scapular prosthesis with a functional latissimus dorsi flap.

**Case presentation:**

Two patients diagnosed with malignant tumour of the scapular region were surgically treated after proper multidisciplinary evaluation. The first patient underwent the procedure as a first surgery, the second as revision surgery. The new technique comprises three surgical stages: excisional surgery with soft tissue resection and scapulectomy, bone reconstruction with custom-made prosthesis, and soft tissue reconstruction using a latissimus dorsi rotational flap overturned on the prosthesis. The goal is to set up a new functional unit combining an anatomically shaped implant (manufactured using latest three-dimensional printing technology) and a muscular flap, and to maintain the neurovascular supply. The patients were followed up to evaluate functional outcome and complications. Both patients were alive with no evidence of disease. Functional results were satisfactory and the Musculoskeletal Tumor Society scores were 87% and 63%, respectively. No surgical complications such as implant breakage, joint collapse, wound dehiscence, or infection were observed.

**Conclusions:**

This new technique upgrades the role of the latissimus dorsi flap to a functional tool in combination with an anatomical, three-dimensionally printed, custom-made prosthesis, and provides adequate well-vascularized and healthy tissue to maximize the likelihood of successful limb salvage.

## Background

Sarcomas that arise from the scapula or periscapular soft tissue present a difficult diagnosis. These tumours often grow quite large before diagnosis, and during disease progression the extension of the tumour to the chest wall, the proximal humerus, the rotator cuff, or the neurovascular bundle may occur. Thus, when a complete surgical resection is possible, a total scapulectomy is the treatment of choice in the majority of patients. In other patients, a specific shoulder-girdle resection can be selected from the six types that were well classified by Malawer [1]. Today, one of the main goals of reconstructive surgery is to preserve adequate function for the basic activities of daily life. This implies adequate management of both bone and soft tissue. Although various methods have been developed, no optimal procedure has emerged [2]. The conventional approach provides no reconstruction of the scapula with humeral suspension to the clavicle or arthrodesis to chest wall. This technique has evolved into a total shoulder replacement incorporating prosthetic devices for both the scapula and the proximal humerus [3]. This definitely allows a fair functional and cosmetic improvement, but failure and infection of the implants still remain a problem. Additionally, allograft reconstruction or autologous recycled bone graft reconstruction (using extracorporeal irradiation, autoclaving, pasteurization, or freezing) have revealed deficits, including fractures and bone resorption [4]. In the last few years, custom-made prosthetic implants seemed promising, but their success is closely linked to the management of the soft tissues, which always play a crucial role [5].

Oncologic resection around the shoulder often results in large complex tissue defects that do not allow primary closure or preservation of function. Significant soft tissue removal is associated with many complications, including infection, which is enhanced by the introduction of foreign material, long operative time, and wide exposure, as well as loss of shoulder movement due to the resection of important muscle masses and contracture that develop during healing. Many reconstructive procedures have been described, and techniques vary from free tissue grafting to local or free flap. With the largest surface area of any extremity-related muscle in the body, the latissimus dorsi (LD) flap is the most widely used as it can cover most large defects of the shoulder [6, 7]. Moreover, the vascular pedicle which utilizes the thoracodorsal artery from the subscapular system allows extensive mobilization. It can be used as a pure muscle flap or with a skin paddle, and when the innervation is properly saved, it can be used to restore some movement of the shoulder.

## Case presentation

**Fig. 1:**
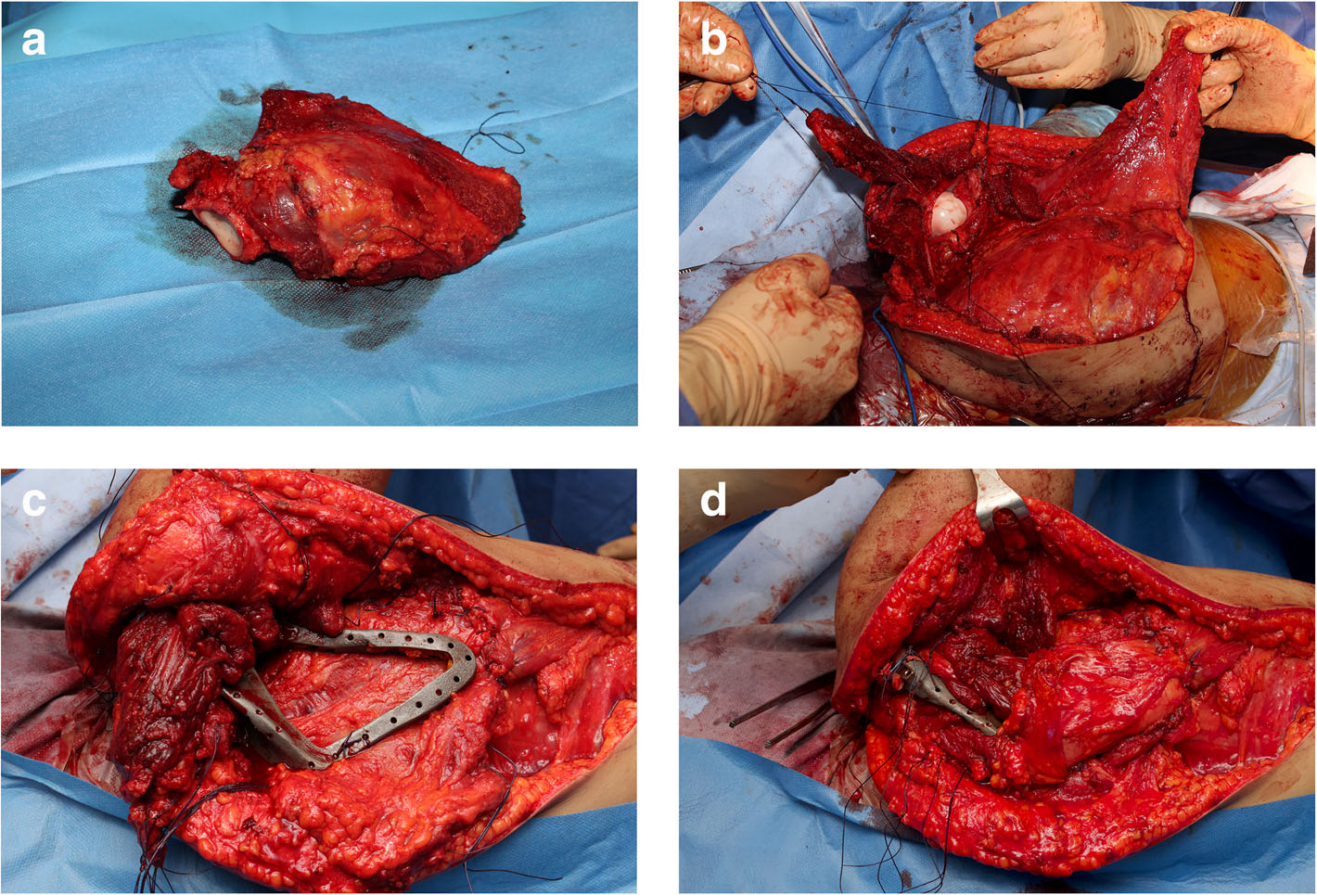
**a** The surgical technique involves excision of the tumour by a total scapulectomy and surrounding soft tissue resection, **b** elevation of a latissimus dorsi (LD) vascularized flap, **c** implant of a custom-made scapular prosthesis, and (**d**) coverage of the implant by the overturned LD flap, creating a “muscular pocket”

We present two patients operated on with a new surgical technique that combines a functional LD flap with a custom-made prosthesis of the scapula after oncological wide resection. This novel surgical technique is performed in three surgical stages: excisional surgery with soft tissue resection and scapulectomy, bone reconstruction, and soft tissue reconstruction (Fig. 1). While under general anesthesia, the patient is placed in the lateral decubitus position with the option for tilting. The hemithorax is prepared and draped, and the entire upper extremity is included in the field. The utilitarian shoulder girdle approach is used with a modified incision turning around the acromioclavicular joint [8]. The axillary vessels and plexus are explored and mobilized anteriorly. The posterior incision permits the release of all muscles attached to the scapula. If the tumour extension allows, the glenohumeral joint is removed intra-articularly to provide better joint reconstruction with the scapular prosthesis. If possible, a portion of the acromion is left intact, both for deltoid muscle reinsertion and better prosthesis anchoring. Moreover, in order to preserve abduction and rotating capacity, it is mandatory to maintain the suprascapular nerve and almost part of the supraspinatus muscle — but obviously, this is secondary to the needs of oncological resection. The infraspinatus muscle and even more of the subscapularis muscle were removed in the majority of our patients, so adequate safety margins could be applied. All of the preserved muscles, tendons, and capsules were marked for later reattachment.

**Fig. 2:**
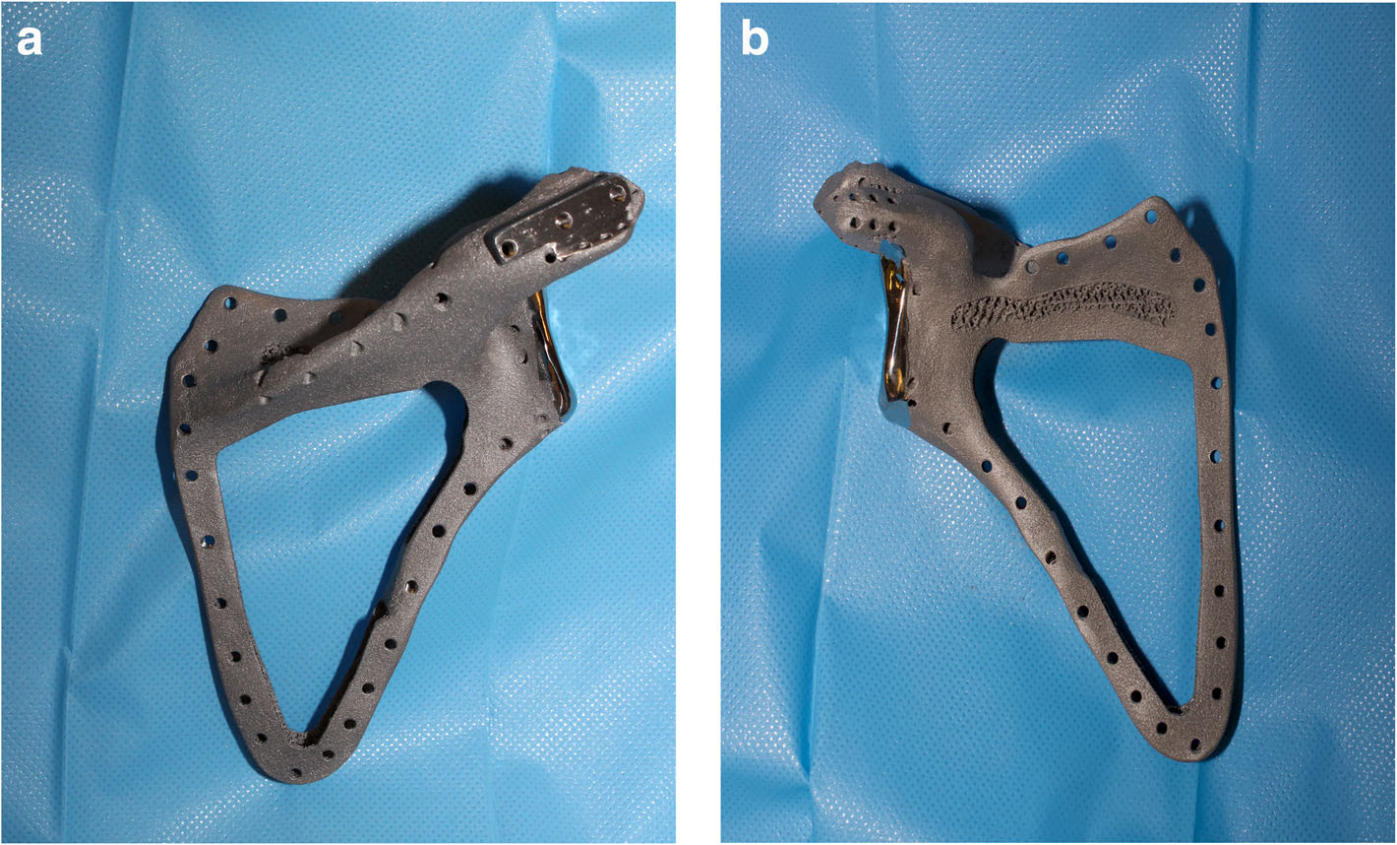
Three-dimensional (3D) design of a custom-made scapular prosthesis on the basis of CT imaging acquisition is shown. The prosthesis was manufactured using 3D printing technology. **a** dorsal view, and (**b**) ventral view

To reconstruct large bone defects, customized prostheses recently have been used after injury or tumour resection and were used in our new technique. The patient’s image data was imported into a computer-aided design system, and a three-dimensional (3D) model of the resected bone was reconstructed (Fig. 2) [5]. The size-matched prosthesis was designed by scaling down data of the contralateral site. Custom manufacturing of the porous titanium implant was then developed. The prosthesis outlines the perimeter of the patient’s scapula with a series of perimeter holes for the reinsertion of soft-tissue structures. The implant was adapted to the remaining portion of the acromion as designed, and it was carefully placed in the correct anatomic position on the chest wall. It was suspended with non-absorbable sutures through suture holes in the residual acromion (if present), which were aligned with the suture holes in the implant. The capsule of the glenohumeral joint was then circumferentially reattached. The humeral head was suspended by suturing the remaining rotator cuff and biceps tendon to the clavicle and neighbouring muscles. Following oncologic resection and bone reconstruction, the extent of the soft tissue wound should be assessed and the soft tissue reconstruction adequately planned [9]. The procedure is usually performed by a plastic surgeon. Extending the same incision up to the distal insertion of LD, a muscular flap is elevated. LD is divided from thoracolumbar fascia, while continuing to raise the flap in an inferior-to-superior direction (towards the humeral insertion and the serratus anterior muscle). Careful dissection is required when approaching the thoracodorsal artery, which provides the vascular supply for the LD. The length and reliability of the thoracodorsal artery makes it possible to mobilize the LD flap as a pedicled flap to the level of the mid-forearm. The thoracodorsal nerve accompanies the vascular pedicle and is often included to minimize atrophy and allow effective muscle contraction. The posterior border of LD is dissected while the humerus attachment remains intact, and careful hemostasis is necessary (particularly in the distal and posteromedial areas where segmental branches of the intercostal arteries additionally supply the muscle). After complete elevation of the muscle and neurovascular pedicle, we perform a unipolar functional transfer by overturning the flap over the prosthesis. The proximal section remains interposed between the prosthesis and chest wall while the distal section provides coverage of the prosthesis. The free end of the flap is sutured to the acromion or to the suture holes of the implant. All of the surrounding residual muscles and tendons (rotator cuff, biceps, and neighbouring muscles) are reattached using sutures to cover the implant. So, a new functional unit is created by the coupling of the custom-made prosthesis and LD flap that wraps it as a muscular functional envelope. To minimize the risk of hematoma or seroma, the wound is closed by direct approximation over a suction drain. For the first 40 postoperative days, patients are immobilized with a brace (at 40° abduction), which is followed by rehabilitation beginning with passive motion up to recovery of an active range of motion.

From September 2016 to January 2017, two female patients with malignant tumours received surgery using the described technique in our department. X-ray, computed tomography (CT), magnetic resonance imaging (MRI), and positron emission tomography scan were performed on all patients to determine the tumour margin, tumour stage, and cutting plane. All patients underwent needle biopsy before surgery to obtain pathological diagnoses. In relation to the planning of LD flap, CT scan and MRI angiography are not routinely part of the preoperative imaging, as the thoracodorsal pedicle is constant and reliable.

Our patients underwent follow up every 3 months for the first 2 years, and then every 6 months thereafter to assess flap survival, functional results, and complications with plain radiographs and a CT scan. Function was evaluated by the Musculoskeletal Tumor Society (MSTS) scoring system [10]. Disease progression follow up was carried out according to oncological protocols.

### Patient 1

**Fig. 3:**
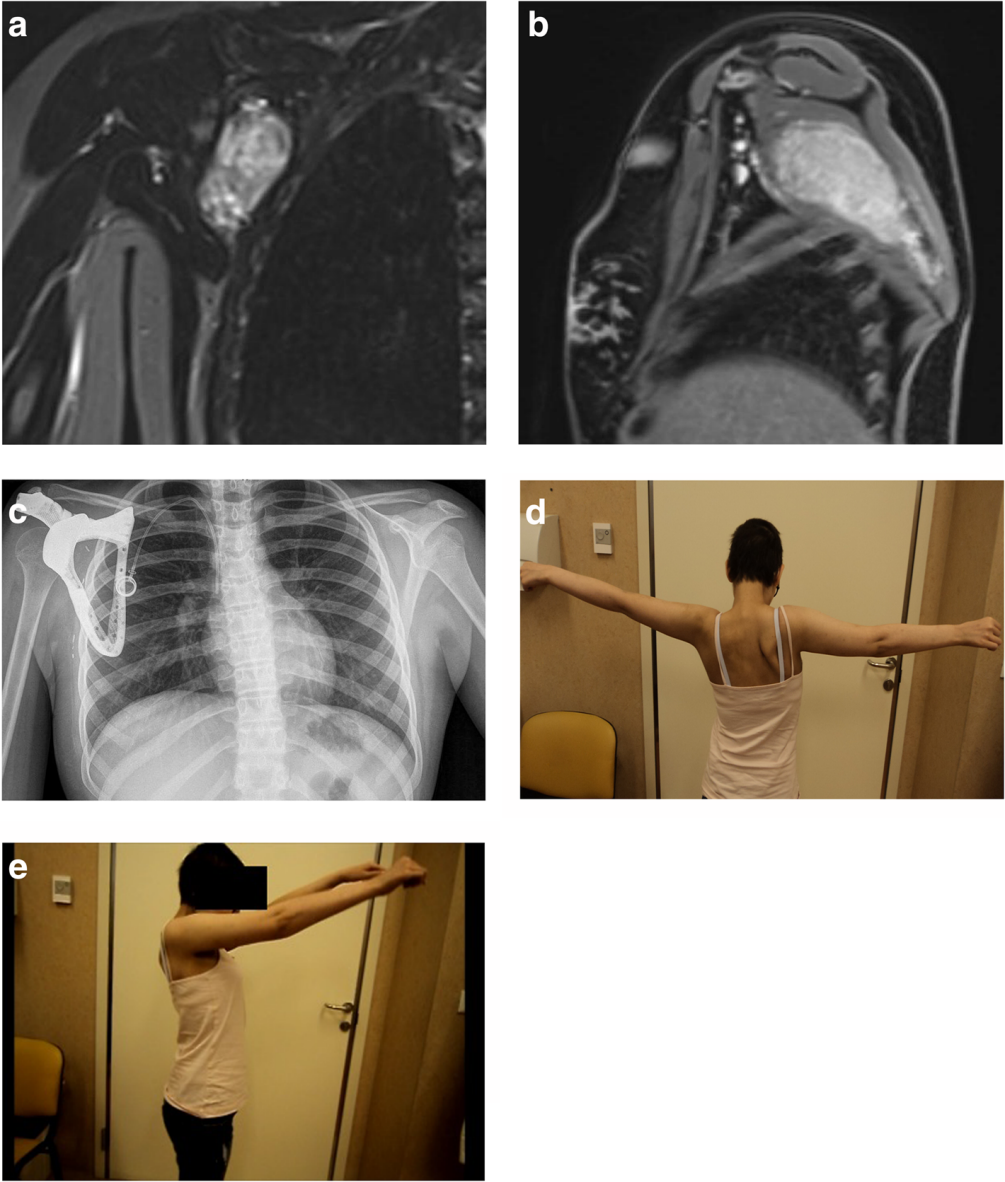
**a** and **b** Patient 1: Preoperative magnetic resonance imaging scan and (**c**) postoperative radiograph show the correct positioning of the custom-made scapular prosthesis. The functional outcome of the procedure is shown. **d** Abduction and (**e**) elevation

An 8-year-old female presented with a history of gradually increasing swelling and pain in the scapular region. The MRI scan showed a large expandable mass involving the subscapularis muscle (Fig. 3). A staging study did not reveal evidence of spread of the disease. The biopsy revealed the diagnosis of extraskeletal Ewing Sarcoma. The patient received neoadjuvant chemotherapy as per the existing hospital protocol. Before surgery, all image data were imported and surgical planning was made on a 3D tumour model. The scapular prosthesis was designed according to images of the contralateral site. During surgery, the lesion was exposed and a subtotal scapulectomy with excision of the involved muscles with a tumour-free margin was completed. The prosthesis was implanted and fixed with nonabsorbable suture to the residual acromion, and created a fundamental fulcrum for the functionality of the system. Residual muscular tissues then were fixed to the small holes on the prosthesis, and the LD flap was created as described above.

### Patient 2

**Fig. 4:**
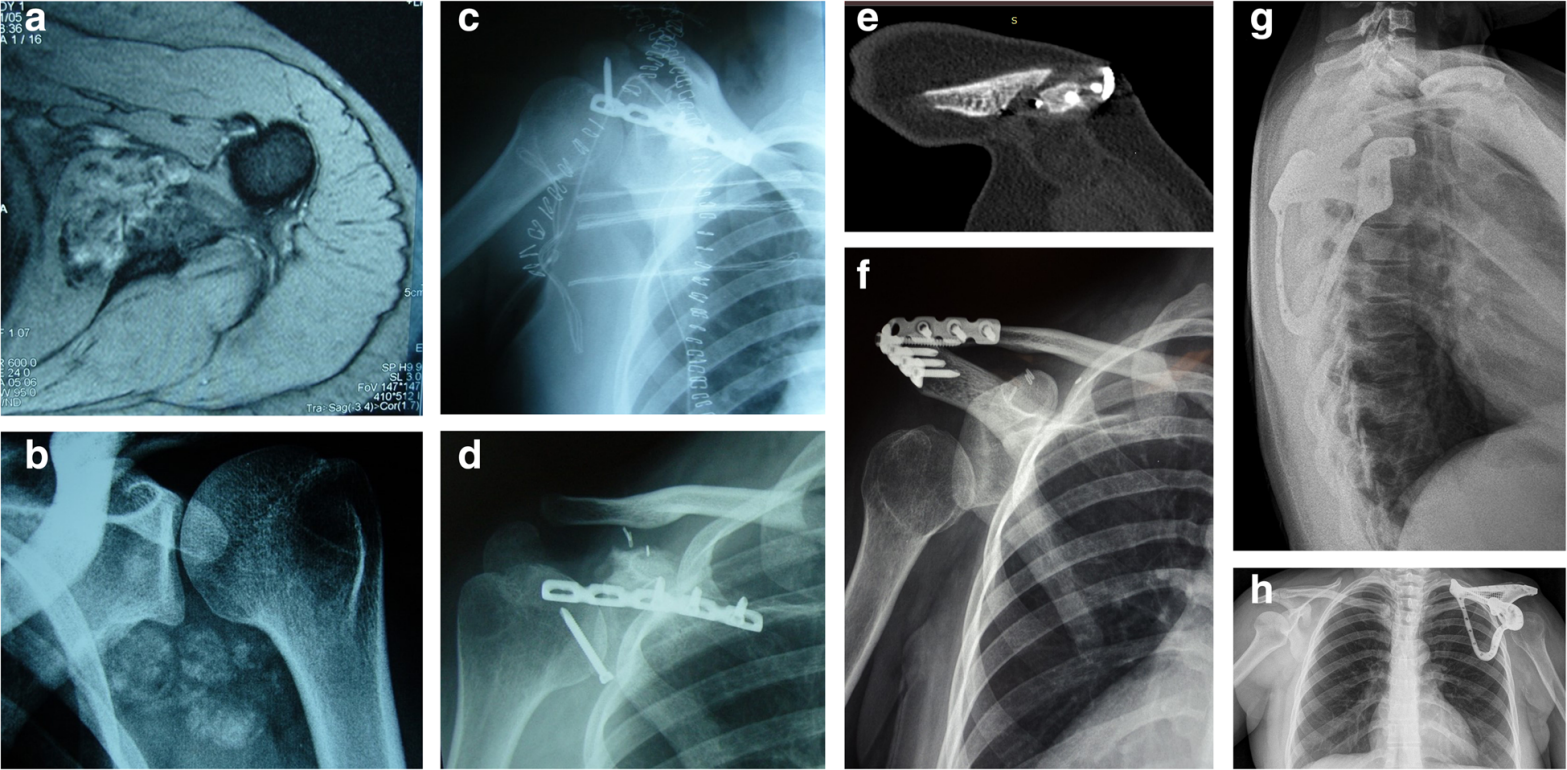
Patient 2: **a** Preoperative magnetic resonance imaging scan and (**b**) radiography of the tumour site. **c** The result of the first surgery (scapular autograft after cryotherapy) and (**d**) following resorption is shown. **e** The result of the second surgery (bone massive allograft) and (**f**) following fatigue fracture are shown. **g** and **h** Radiographic results of the latissimus dorsi rotational flap and custom-made scapular prosthesis implant after a third surgery are shown

A 45-year-old woman was diagnosed with subscapularis muscle synovial sarcoma in 2006 (Fig. 4). After a proper staging study was completed, an autograft of the scapula after cryotherapy with liquid nitrogen was performed. After surgery, the patient received adjuvant chemotherapy. Five years after surgery, massive resorption of frozen graft and osteosynthesis failure were observed, which necessitated revision surgery. The patient underwent reconstruction with massive bone allograft. Another complication occurred 4 years later which involved a fatigue fracture of the acromion with pain and loss of function. We decided to perform a salvage procedure with a custom-made prosthesis, according to the data of the contralateral side. Due to the absence of the acromion, the implant was not anchored to the bone, but only to residual muscles and ligaments. Moreover, the lack of soft tissue because of multiple surgeries makes the LD flap particularly suitable and effective in that case.

### Results

The average operative time of both patients was 337 min (354 and 320 min). There was no neurovascular bundle injury or other complications that occurred during surgery. The follow-up time was 20 and 16 months in Patients 1 and 2, respectively. No local recurrence or metastasis was observed. At their latest follow-up, both patients were alive with no evidence of disease. The range of motion of the shoulders are summarized in Table 1. According to MSTS system for the upper limb, the scores were 87 and 63% in Patients 1 and 2, respectively (Table 2). There was no implant breakage and joint collapse in either patient.

**Table 1:** Final range of motion of study patients

	Flexion	Extension	Abduction	External rotation	Internal rotation
Patient 1	120°	30°	110°	40°	60°
Patient 2	40°	10°	30°	10°	10°

**Table 2:** Clinical outcome at final follow-up^a^

	Pain	Function	Emotional	Hand positioning	Manual dexterity	Lifting ability	Total
Patient 1	5	4	4	4	5	4	26 (87%)
Patient 2	5	2	3	3	5	1	19 (63%)

^a^Measured according to Musculoskeletal Tumor Society scoring system for the upper limb

## Discussion

For malignant tumours of the scapular region, limb salvage surgery has become, in the past years, the consensus procedure for most patients. Wide resection with total scapulectomy is often necessary, implying complex shoulder defects. Residual function is usually unsatisfactory with traditional methods of reconstruction (humeral suspension, recycled bone graft, massive bone allograft, or total shoulder replacement). We have described a new technique that combines custom-made scapular prosthesis and LD rotational flap in an innovative functional unit. For scapular reconstruction, we chose a customized 3D printed titanium prosthesis based on the good results recently observed in the literature [5, 11, 12]. This new device has proved to overcome the issues linked to previous systems. For example, humeral suspension provides poor functionality and unsatisfactory cosmesis. Additionally, although better results are reported with allograft and autograft, there are high rates of complications, such as fracture of the grafted bone and implant fixation failure. With regard to total shoulder replacement, which requires humeral head replacement even in the absence of oncologic lesions, our system permits to retain the humeral head. Moreover, customisation allows retention of scapular joint congruity, prevention of humeral dislocation, and early wear of humeral articular cartilage. Before surgery, we built a 3D model of the shoulder joint using a 3D printer, which significantly helps the surgeon to assess implant fit and evaluate fixation sites. The preoperative planning was well performed, reducing operative time and surgical stress for the patient. The 3D printing technology was also used to build the prosthesis, reducing considerable production time. The material of choice was a titanium alloy, which is widely used in prosthetics. Titanium alloy has good resistance to bending stresses and compressive stresses. Moreover, an undisputed advantage is the excellent capacity for osseointegration, which is fundamental at the interface with residual acromion.

A total scapulectomy requires not only bone and joint resection, but also wide excision of shoulder girdle muscles. Although various soft tissue reconstructions have been applied, an optimal technique has not yet been determined particularly because of the lack of and variability of remaining muscle and rotator cuff following excision surgery. Reconstruction techniques should recreate the anatomical contour of the shoulder girdle, require durable and stable coverage of graft and/or prosthetic constructs, and strive to preserve the wide range of motion of the shoulder. The technique we reported aims to find a solution to these problems and aims to reach these goals. The crucial point is the overturning of the flap over the prosthesis, creating a kind of “muscular pocket” (Fig. 2). The role it plays is manifold and innovative:

- It provides adequate coverage of the implant, and constitutes the most effective biologic barrier against infections. Moreover, the muscular tissue interposed between the implant and the skin prevents superficial dehiscence, improves the healing of the surgical wound, and offers an anatomical profile to the shoulder.
- It is helpful in sustaining the implant, allowing an “in-site position” of the customized scapula and improving the efficacy of the surrounding sutures.
- The interposition of a muscular plane between the prosthesis and chest wall constitutes an effective sliding plane for the scapulo-thoracic joint, avoiding chest irritation phenomenon or osteopathic processes of the ribs.
- The preserved innervation of LD ensures functionality to the muscular flap. It is able to assist movement, providing a flap/prosthesis functional uniqueness. Moreover, the contraction of LD muscle permits dynamic stabilisation of the implant.

The patients in our series obtained benefits from this new technique of reconstruction. Firstly, no complications were observed (infection, wound problems, instability, or failure of the implant). This is in contrast to the complications Patient 2 experienced prior to our surgery. She endured various types of adverse events during the previous 10 years related to other reconstruction (recycled bone autograft and massive bone allograft) she underwent at other institutions.

The oncological outcome was good in both patients with no progression of disease and the patients sustained a good state of general health. Functionality was good in Patient 1 (MSTS score 87%) and acceptable in Patient 2 (MSTS score 63%) [5, 11, 12]. This discrepancy is easily explained. Patient 1 underwent the procedure as a first surgery while the second patient had several failures previously. The weakness of residual tissue and scars considerably influenced the results. Moreover, there was a technical difference among the patients: the prosthesis was sutured to a residual portion of the acromion in Patient 1 while it was completely suspended through soft tissue sutures in Patient 2 (because the acromion was completely removed at the first surgery in 2006). This point of contact between the bone and implant, together with the remaining acromion-clavicular articulation, acts as a fulcrum. This assists scapular movement and seems to improve the overall functionality. The described technique also seems promising in the pediatric age group. Children and adolescents in whom this surgery can be addressed have high functional requests and bone grafts do not offer adequate long-lasting mechanical guarantees [13]. Even conventional prostheses are exposed to risks of early wear and failure in this population. However, to our knowledge, there has been no previous report of custom-made prosthesis in pediatric patients. Potential drawbacks are linked to the technical features of the prosthesis, such as the time required for osseointegration, the limited long-term durability of the implant (especially for mechanical failures), and articular degeneration at the interface between prosthesis and cartilage of humeral head.

Our study had several limitations. It was limited by the small sample of patients and short-term follow-up. It was difficult to obtain a large number of patients in a single institution due to the rarity of the pathology. The small sample size did not allow sufficient power to explore the advantages of this techniques and eventual complications. The authors can only provide the preliminary results to introduce this technique in the current study.

## Conclusions

In conclusion, custom-made prosthesis designed from patient-imaging data demonstrated good reliability even in scapular reconstruction. But when oncologic resection presents a reconstructive challenge, stable and durable soft tissue coverage is critical. The use of a pedicled LD flap provides adequate well- vascularized and healthy tissue to maximize the chances of successful limb salvage and minimize the risks of wound problems and deep infection. And moreover, this new technique we introduced upgrades the role of LD flap to functional tool in association with 3D printed custom-made prosthesis.

## Abbreviations

3D: Three-dimensional
CT: Computed tomography
LD: Latissimus dorsi
MRI: Magnetic resonance imaging
MSTS: Musculoskeletal Tumor Society
